# Gonadotropin therapy in idiopathic hypogonadal non-obstructive azoospermia (APHRODITE Groups 3–4): a multicenter randomized controlled trial

**DOI:** 10.3389/frph.2026.1867412

**Published:** 2026-07-09

**Authors:** Vipin Chandra, Sandro C. Esteves, Shashank Sanagoudar

**Affiliations:** 1 Department of Clinical and Lab Operations, Indira IVF Hospital Limited, Udaipur, India; 2 Department of Urology and Reproductive Medicine, Androfert Centro de Referencia de Reproducao Masculina SC Ltda, Campinas, Brazil

**Keywords:** gonadotropin therapy, idiopathic hypogonadal, non-obstructive azoospermia, micro-TESE, APHRODITE criteria, male infertility, standard of care management

## Abstract

**Introduction:**

Idiopathic non-obstructive azoospermia (NOA) is a severe form of male infertility with limited non-surgical treatment options. Microdissection testicular sperm extraction (micro-TESE) remains the standard approach for sperm retrieval, yet success rates vary substantially, and many patients undergo surgery without successful sperm recovery. Hormonal optimization using human chorionic gonadotropin (hCG) with or without follicle-stimulating hormone (FSH) has been proposed to improve spermatogenesis in hypogonadal men, but evidence from randomized controlled trials is lacking. This multicenter trial evaluates whether gonadotropin-based hormonal optimization improves sperm availability for intracytoplasmic sperm injection (ICSI) compared with standard-of-care (SOC) management in men with idiopathic NOA and biochemical hypogonadism.

**Methods and analysis:**

This multicenter, randomized, controlled, parallel-group superiority trial will allocate participants 1:1 to hormonal therapy or SOC. Eligible participants are men with idiopathic NOA, total testosterone <350 ng/dL on two fasting morning tests, and FSH ≥7.6 IU/L, consistent with APHRODITE Groups 3–4. Key exclusion criteria include cryptorchidism, prior chemotherapy or radiotherapy, defined genetic NOA (e.g., AZFa/complete AZFb deletions), prior micro-TESE, recent hormonal therapy use, uncontrolled endocrine disease, severe liver disease, polycythemia, active malignancy, inability to comply with the study procedures, and clinically significant varicocele. Participants assigned to the intervention arm will receive gonadotropin-based hormonal optimization using hCG and recombinant FSH, with hormonal monitoring and hCG dose titration throughout treatment. The control arm will receive standard management without hormonal therapy. The primary endpoint is a composite treatment-policy outcome defined as sperm availability suitable for ICSI at any time from randomization through Week 16, either through ejaculated sperm or through micro-TESE. Secondary outcomes include micro-TESE sperm retrieval success rate, need for surgery, embryologic outcomes, reproductive outcomes, and treatment safety. The study is designed as a pragmatic comparative effectiveness trial that intends to evaluate whether biologically informed hormonal optimization improves clinically meaningful sperm availability under routine clinical practice conditions.

**Ethics and Dissemination:**

The study was approved by Institution Ethics Committee (IIVF-UA00l 00).

**Clinical Trial Registration:**

(https://clinicaltrials.gov/study/NCT07540611), identifier NCT07540611.

## Introduction

Non-obstructive azoospermia (NOA) represents the most severe form of male infertility and affects approximately 10%–15% of infertile men (1). In men with idiopathic NOA, impaired intratesticular testosterone production and elevated follicle-stimulating hormone (FSH) levels often reflect underlying Sertoli cell dysfunction and reduced spermatogenic capacity (2). Currently, microdissection testicular sperm extraction (micro-TESE) remains the preferred method of sperm retrieval in these patients, offering the highest likelihood of obtaining sperm for intracytoplasmic sperm injection (ICSI). However, sperm retrieval success rates remain highly variable, generally ranging from 40% to 60%, and many men undergo invasive surgery without successful sperm recovery (3, 4).

Despite extensive investigation, reliable preoperative predictors of sperm retrieval remain inconsistent across studies and populations. Several clinical, hormonal, and molecular biomarkers have been explored, including FSH, inhibin B, anti-Müllerian hormone, and testicular histopathology, but no single marker has demonstrated sufficient predictive accuracy for routine clinical use. Representative studies evaluating biomarkers and predictors of sperm retrieval success in idiopathic NOA are summarized in Table 1.

**Table 1:** Clinical studies involving predictors/biomarkers for sperm retrieval in idiopathic NOA and related micro-TESE outcomes.

Year	Reference	N	Population	Sample type	BM(s)	Threshold (cutoff)	Sens	Spec
2023	Deng et al. (14)	168	Idiopathic NOA undergoing micro-TESE	Serum	INHB; AMH; INHB/AMH	INHB 21.51 pg/mL; INHB/AMH 3.19	86.3% (INHB/AMH)	53.8% (INHB/AMH)
2021	Zarezadeh et al.(17)	—	NOA (predictors review)	Serum/seminal (review)	FSH, LH, TT, INHB, AMH, E2, PRL, leptin (various)	Heterogeneous; no unified cutoffs	—	—
2021	Majzoub et al. (with S.C. Esteves) (16)	297	NOA; staged Testicular Sperm Aspiration (TESA) → micro-TESE	Clinical/serum	Multivariable model (age, testis size, FSH, etc.)	Model-based (no single threshold)	NR	NR
2021	Shiraishi et al. (18)	76	NOA vs. OA; subset on anastrozole	Testicular fluid/serum/tissue	Intratesticular T and E2, aromatase expression	No diagnostic cutoff; mechanistic	—	—
2023	Shi et al. (15)	114	Idiopathic NOA, micro-TESE	Testicular tissue	circ_MGLL (circRNA) + clinical nomogram	Nomogram (probability-based)	-	-
2021	Achermann et al. (Review) (1)	4,895 (across 116 studies)	NOA; micro-TESE effectiveness and complications	—	—	SRR ∼46.6% overall; naïve ∼46.8%	—	—
2024	Mahdy et al. (2)	172	NOA, micro-TESE	Clinical/serum/histology	Histology; clinical factors	NR	—	—

BM, biomarker; INHB, inhibin B; AMH, anti-Müllerian hormone; TT, total testosterone; Spec, specificity; Sens, sensitivity; NPV, negative predictive value; NR, not reported.

Emerging evidence suggests that optimization of the hypothalamic–pituitary–gonadal axis, particularly correction of hypogonadism prior to surgical sperm retrieval, may enhance spermatogenic output and potentially improve the likelihood of extracting sperm at surgery (3). However, robust randomized controlled data assessing the efficacy of gonadotropin therapy in idiopathic NOA are lacking.

To improve phenotypic stratification and guide therapeutic decision-making, Esteves et al. proposed the APHRODITE criteria, a clinically oriented classification system based on patient characteristics, semen profile, and readily available hormonal parameters, particularly FSH and total testosterone (TT) levels (5).

The APHRODITE criteria were specifically developed to identify subgroups of men with idiopathic infertility and/or hypogonadism in whom endocrine modulation may be endocrinologically and clinically relevant (5).

Within this scheme, men classified as APHRODITE Groups 3 and 4 exhibit biochemical hypogonadism associated with within normal ranges or elevated FSH levels, suggesting partial but potentially recoverable testicular dysfunction. In these individuals, physiologic restoration of the hypothalamic–pituitary–gonadal axis using human chorionic gonadotropin (hCG), with or without adjunctive recombinant FSH, has been proposed as a strategy to improve intratesticular hormonal support, enhance Sertoli cell function, and potentially stimulate residual spermatogenesis (6). Observational studies and systematic reviews have suggested a possible association between hormonal optimization and improved sperm retrieval outcomes in selected NOA populations (1, 6, 7), although high-quality randomized evidence remains lacking.

To address this critical evidence gap, we designed a multicenter, randomized, controlled superiority trial comparing a structured gonadotropin-based hormonal optimization strategy versus standard-of-care (SOC) management in hypogonadal men with idiopathic NOA classified within APHRODITE Groups 3 and 4. The trial evaluates whether hormonal therapy improves sperm availability for ICSI within a 16-week treatment window, either through ejaculated sperm or micro-TESE. Importantly, the study was designed as a pragmatic comparative effectiveness trial rather than a placebo-controlled efficacy study, aiming to determine whether a biologically informed hormonal optimization strategy improves clinically meaningful sperm availability for ICSI under routine clinical practice conditions.

## Method and analysis

### Study objective

The primary objective of this multicenter randomized controlled trial is to determine whether physiologic gonadotropin-based hormonal optimization (hCG with FSH) increases the likelihood of obtaining sperm suitable for ICSI—either through ejaculated sperm or through micro-TESE—within 16 weeks in hypogonadal men with idiopathic NOA, compared with SOC management without hormonal therapy.

The secondary objective is to evaluate the impact of hormonal therapy on surgical sperm retrieval rates, need for surgery, embryologic outcomes (fertilization, blastulation, blastocyst quality), and reproductive outcomes including clinical pregnancy, miscarriage, and live birth, as well as to assess treatment safety.

We primarily hypothesize that gonadotropin-based hormonal optimization (hCG + FSH) will increase the proportion of men with idiopathic NOA who achieve sperm availability for ICSI—either through ejaculated sperm or through micro-TESE—by Week 16, compared with standard-of-care management.

Our secondary hypothesis is that hormonal therapy will reduce the need for micro-TESE compared with standard-of-care and improve surgical sperm retrieval rates among men undergoing micro-TESE.

### Population

The inclusion criteria are as follows: idiopathic NOA; hypogonadal (TT <350 ng/dL on two fasting morning tests); and FSH ≥7.6 IU/L (APHRODITE Group 3: 7.6–12.0 IU/L; Group 4: >12.0 IU/L). The exclusion criteria are as follows: cryptorchidism, chemo/radiation, genetic NOA (e.g., AZFa/complete AZFb), testicular trauma/torsion, postorchitis; prior micro-TESE within 12 months; recent gonadotropin therapy (<6 months); uncontrolled endocrine disease; active malignancy; severe liver disease; polycythemia (Hct > 50%); inability to comply; and varicocele ≥Grade 3.

### Study design and methods

This multicenter, randomized, controlled, parallel-group superiority trial assigned men 1:1 to hormonal therapy or SOC. Eligible participants had idiopathic NOA, total testosterone <350 ng/dL on two fasting morning tests, and FSH ≥7.6 IU/L (APHRODITE Groups 3–4). Key exclusions included cryptorchidism, prior chemotherapy/radiation, genetic NOA (AZFa/complete AZFb deletions), prior micro-TESE within 12 months, recent gonadotropin use, uncontrolled endocrine disease, severe liver disease, polycythemia, active malignancy, inability to comply, and presence of untreated clinical varicocele requiring correction. Table 2 illustrates the summary of the study design.

**Table 2:** Summary of study design.

Design	Multicenter, randomized, controlled, parallel-group superiority trial (1:1)
Population	Idiopathic NOA; hypogonadal (TT <350 ng/dL on two fasting morning tests); FSH ≥7.6 IU/L (APHRODITE Group 3: 7.6–12.0 IU/L; Group 4: >12.0 IU/L). Exclusions: cryptorchidism, chemo/radiation, genetic NOA (e.g., AZFa/complete AZFb), testicular trauma/torsion, postorchitis. prior micro-TESE within 12 months; recent gonadotropin therapy (<6 months); uncontrolled endocrine disease; active malignancy; severe liver disease; polycythemia (Hct > 50%); inability to comply. Varicocele ≥ Grade 3
Interventions	Arm A: hCG + FSH therapy with monthly hormone-driven titration (hCG initial ∼83 µg SC twice weekly; no preset min/max; target TT >350–900 ng/dL) + FSH 150 IU SC twice weekly (increase to 150 IU SC three times weekly if ‘FSH reset’ < 1.5 IU/L); allow anastrozole 1 mg PO daily/letrozole 2.5 mg half-tablet alternate day if T/E < 10. Arm B: Standard-of-care (SOC) no gonadotropins
Follow-up	Participants in both arms will be followed up from randomization through Week 16 for ascertaining the primary composite endpoint. The SOC arm may undergo micro-TESE at any time within this window per standard scheduling; a semen analysis will be performed on the day of surgery to capture rare ejaculated sperm. The hormonal arm will undergo semen analyses at Weeks 12 and 16, with micro-TESE at Week 16 if ejaculated sperm is absent. No artificial delay is imposed on the SOC arm
Primary outcome	Success is defined as sperm available for ICSI at any time from randomization through Week 16 via ejaculation or micro-TESE, centrally adjudicated, and blinded to allocation
Secondary outcomes	Micro-TESE SSR; need for surgery; safety/harms; ICSI fertilization; blastulation rate; blastocyst quality; top-1quality blastocyst rate; clinical pregnancy rate; miscarriage rate; live birth (exploratory)
Randomization/concealment	Central web-based randomization; permuted variable blocks; stratified by site and APHRODITE group; allocation concealed
Masking	Blinded lab personnel/embryologists and central adjudicators; surgeons/participants unblinded
Estimand	Treatment-policy estimand per ICH E9(R1) for composite outcome up to Week 16. To address potential bias from differential procedure timing, the primary analysis uses a fixed landmark at Week 16 (treatment policy). A time-to-event secondary analysis (time to first sperm availability) will be performed to assess consistency
Semen test on surgery day	Control arm performs a semen analysis on the day of micro-TESE to capture rare ejaculation successes
Interim	Safety-only interim at approximately 50% randomized (≥12-week follow-up); no efficacy/futility testing
BSSR	Blinded sample size re-estimation when ≥30% have completed Week-16 outcomes; adjust total N for nuisance event rate (cap +20%)

**Figure 1:**
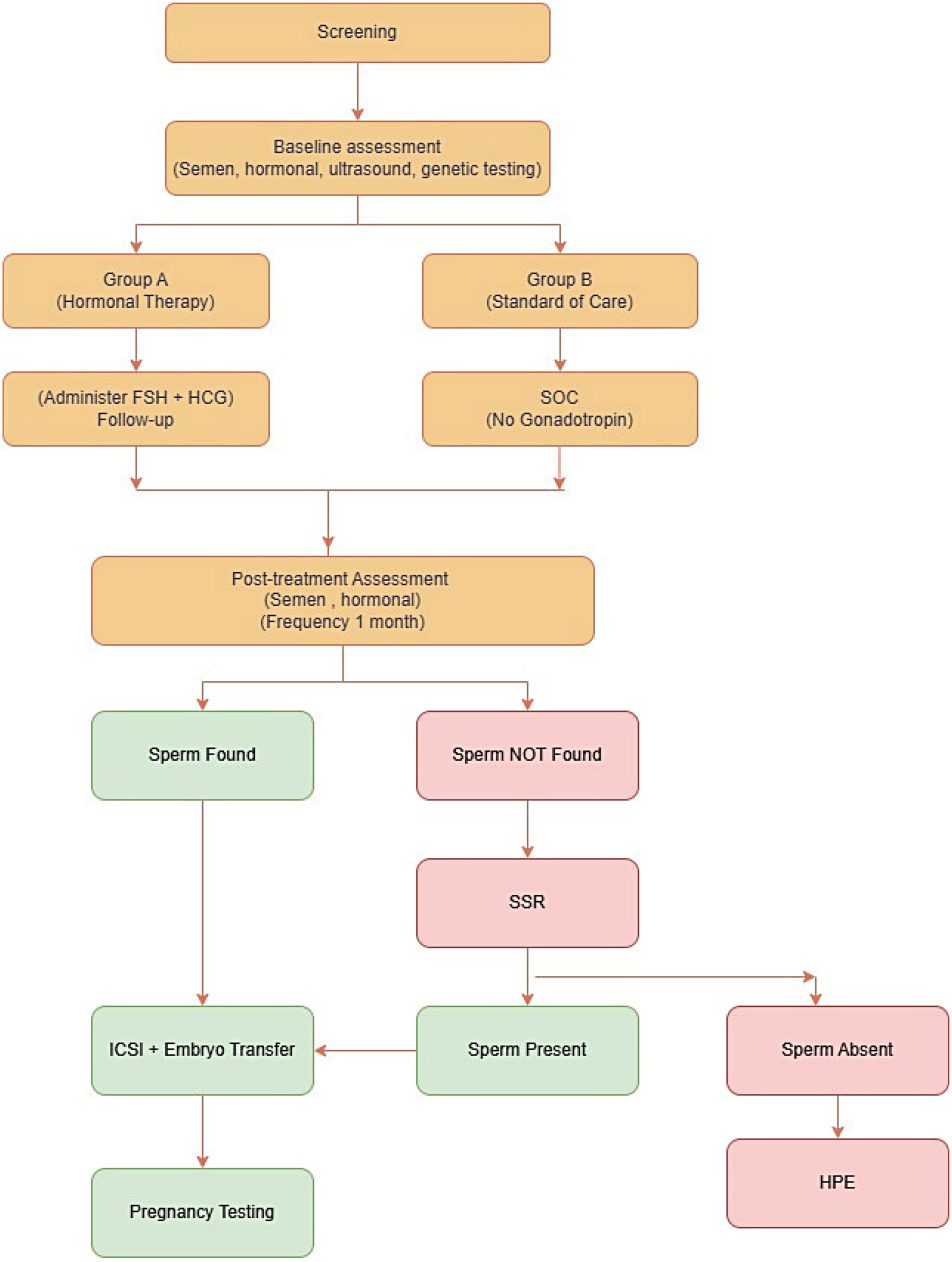
Flowchart for NOA Protocol; FSH, follicle-stimulating hormone; HCG- SOC, standard of care, HCG, human chorionic gonadotropin; SSR, surgical sperm retrieval, HPE, histopathological examination.

The trial flowchart is provided in Figure 1.

### Interventions

**Arm A (Hormonal therapy):**

- hCG initial dose: ∼83 µg SC twice weekly; monthly titration guided by hormone levels with no preset min/max to maintain TT >350–900 ng/dL.
- Monthly endocrine panel: TT, E2, LH, and FSH (plus CBC/Hct, LFTs, lipids) for titration and safety.
- FSH: 150 IU SC twice weekly (fixed); escalation to 150 IU SC three times weekly if “FSH reset” was observed (FSH <1.5 IU/L). The threshold of FSH <1.5 IU/L was selected to identify excessive suppression of endogenous pituitary FSH secretion during hCG therapy. This threshold was physiologically derived from evidence demonstrating that profound FSH deficiency adversely affects Sertoli cell function and spermatogenesis, particularly in hypogonadotropic states (8–10). In addition, observational data from hypogonadal NOA men receiving hCG-based hormonal therapy demonstrated that approximately 20% of patients developed profound endogenous FSH suppression below this threshold during hCG stimulation, which normalized after escalation of exogenous recombinant FSH therapy (11–13).
- Aromatase inhibitor: anastrozole 1 mg PO daily/letrozole 2.5 mg half-tablet alternate day if T(ng/dL)/E(pg/mL) < 10; reassess monthly and taper if normalized.
- Week 12 semen analysis: if ejaculated sperm adequate for ICSI → success; cryopreserve; treatment may stop; otherwise, continue to Week 16.

**Arm B (SOC):** No gonadotropin therapy; micro-TESE could occur anytime within 16 weeks.

### Follow-up

Hormonal therapy participants underwent semen analyses at Weeks 12 and 16, with micro-TESE at Week 16 if no ejaculated sperm were present. SOC participants underwent semen analysis on the day of micro-TESE to detect rare ejaculated sperm. All participants were followed up through Week 16.

### Outcomes

The primary outcome is a composite treatment-policy endpoint defined as sperm availability suitable for ICSI at any time from randomization through Week 16, regardless of whether sperm is obtained through ejaculated semen or through micro-TESE. Participants achieving ejaculated sperm suitable for ICSI before Week 16 will be considered primary endpoint successes and do not require mandatory micro-TESE. In the SOC arm, micro-TESE performed at any time within the 16-week observation window contributes to endpoint ascertainment.

Sperm considered “suitable for ICSI” was defined as the identification of at least one viable spermatozoon considered suitable for intracytoplasmic sperm injection by experienced embryology personnel according to standardized laboratory procedures.

Because the primary endpoint represents a clinically pragmatic composite outcome evaluated under a treatment-policy estimand scheme, the principal analysis prioritizes preservation of randomization and estimation of real-world treatment effectiveness rather than mechanistic isolation of individual procedural pathways.

Secondary outcomes include micro-TESE sample size re-estimation (SSR), need for surgery, safety/harms, ICSI fertilization, blastulation rate, blastocyst quality, top-quality blastocyst rate, clinical pregnancy rate, miscarriage rate, and live birth.

Embryologic and reproductive outcomes are considered exploratory because they are influenced by both male and female factors and depend on successful progression to Assisted Reproductive Technology (ART) treatment.

### Statistical approach

A treatment-policy estimand [ICH E9(R1)] evaluated the composite success at Week 16. A time-to-event analysis of time to first sperm availability served as a secondary sensitivity analysis.

### Sample size estimation

The sample size calculation assumed a control-group success rate of approximately 40%, based on contemporary literature evaluating sperm retrieval outcomes in idiopathic NOA (1, 7). The study was powered to detect an absolute improvement of approximately 10 percentage points in the primary composite endpoint with two-sided *α* = 0.05 and 80%–90% statistical power using a 1:1 allocation ratio. The planned total sample size also accounted for anticipated attrition, intercenter variability, and preservation of reasonable power for the prespecified interaction analysis between APHRODITE Groups 3 and 4.

### Interim analysis and sample estimation

A single planned interim review will be conducted after approximately 50% of participants have been randomized and have completed at least 12 weeks of follow-up. Because the primary endpoint incorporates sperm availability assessed up to Week 16, including micro-TESE outcomes, formal early efficacy or futility analyses based on conditional power were considered inappropriate and were therefore not planned.

Accordingly, the interim review will focus exclusively on

- participant safety,
- protocol adherence, and
- data completeness and trial conduct.

No formal hypothesis testing for efficacy or futility will be performed during the interim review, and no alpha spending is planned.

In addition, a blinded sample size re-estimation (BSSR) will be performed once at least 30% of participants have completed Week-16 outcome assessment. The re-estimation will be based exclusively on the pooled overall event rate for the primary composite endpoint, without unblinding treatment allocation. Any increase in total sample size will be capped at 20% of the originally planned enrollment.

Missing primary endpoint data will be conservatively imputed as treatment failures regardless of reason, consistent with the treatment-policy estimand framework.

Supportive analyses for secondary outcomes will use multiple imputation under the missing-at-random assumption, incorporating treatment arm, study site, APHRODITE group, age, BMI, baseline hormonal profile, testicular volume, and interim semen findings. Sensitivity analyses using delta-adjusted missing-not-at-random assumptions will also be performed.

For adverse events and serious adverse events, missing data will be treated as absence of an event unless contradicted by site monitoring or Data Safety and Monitoring System (DSMB) review.

### Sensitivity analyses

- Per-protocol (PP): exclude major deviations (e.g., elective early micro-TESE at 12 weeks without Week-16 assessment, prohibited concomitants, critical non-adherence).
- Hypothetical estimate: multiple imputation assuming completion to 16 weeks to test robustness to intercurrent events and missingness.
- Inverse population weighty (IPW) sensitivity: for micro-TESE SSR, weight by inverse probability of undergoing surgery to address differential uptake by arm.
- Time-to-availability (ejaculated semen or micro-TESE) up to Week 16: Kaplan–Meier/Cox as supportive analysis.

### Subgroup and heterogeneity analysis plan

The primary subgrouping is based on baseline FSH level according to the APHRODITE classification:

- Group 3: FSH 7.6–12.0 IU/L
- Group 4: FSH >12.0 IU/L

The purpose of a subgroup analysis is to assess whether the treatment effect on the primary endpoint—sperm available for ICSI by Week 16—differs between APHRODITE Groups 3 and 4.

#### Statistical model and test of interaction

The analysis will be interaction-driven. Treatment effects within subgroups will be descriptive unless the treatment-by-subgroup interaction is statistically significant.

A generalized linear model (identity link, robust standard errors) shown in Equation 1 will be used:

$$Y\_i\;=\beta_{0\;}+\beta_{1}\cdot \mathrm{Treatment}\_i+\beta_{2}\cdot \mathrm{Group}\;4\_i+\beta_{3}\cdot (\mathrm{Treatment}\_i\times \mathrm{Group}\;4\_i)+\gamma^{T}X\_i+u\_\mathrm{site}+\varepsilon \_i,$$ (1)

where Y_i represents the binary composite outcome (sperm available for ICSI by Week 16), Group 4_i is an indicator variable for APHRODITE Group 4, and X_i denotes prespecified covariates (female age, male age, center, baseline semen parameters, baseline TT/E2, baseline DNA Fragmentation Index (DFI), and number of Metaphase II (MII)). The interaction term *β*_3_ tests for a differential treatment effect between Groups 3 and 4. Random intercepts for site will account for clustering, and results will be presented as marginal risk differences.

The test for interaction will use a two-sided *α* = 0.05. Although prospectively specified and biologically motivated, subgroup analyses are considered supportive and hypothesis-generating unless sufficiently large interaction effects are observed. The trial is primarily powered for the overall treatment effect rather than formal interaction testing. The Table 3 illustrates SPIRIT schedule for enrollment, intervention, and assessments.

**Table 3:** SPIRIT schedule of enrollment, interventions, and assessments.

Procedure/time point	Enrollment (≤28 days)	Baseline (W0)	W12	W16
Eligibility and consent	X			
Randomization		X		
hCG + FSH (Arm A)		X	X	X
Endocrine labs (TT, E2, LH, FSH; CBC/Hct, LFTs, lipids)		X	X	X
Semen analysis		X	X	X
Micro-TESE (if needed)				X
AE/SAE assessment		X	X	X

#### Reporting and visualization

Forest plots will display risk differences (treatment − control) with 95% confidence intervals for the overall population and for each subgroup. The interaction *p*-value will accompany the forest plot. A Supplementary Figure will show treatment effect as a function of continuous FSH modeled with restricted cubic splines (three to four knots) and 95% confidence bands.

#### Multiplicity control

This is the only prespecified effect-modification test. All other subgroup analyses (e.g., testosterone level and age strata) are exploratory and will be labeled accordingly. No alpha adjustment is required provided only this single interaction is tested. If additional interactions are introduced, family-wise error rate will be controlled using the Holm procedure.

#### Sensitivity analyses

1. Continuous FSH model using restricted cubic splines.
2. Mixed-effects logistic regression with logit link; marginal effects reported as risk differences.
3. Per-protocol and as-treated analyses repeating the interaction test.
4. Center robustness: treatment ×   subgroup ×   center term explored if strong site heterogeneity is suspected.

#### Power considerations

Subgroup and interaction analyses are less powered than main effects. With approximately 860 participants in total (≈425 per arm) and an SOC success rate of 40%, the study will have sufficient power to detect large interactions (≥10 percentage-point difference in treatment effects between Groups 3 and 4). Smaller differences will be interpreted cautiously and only in the context of consistent secondary outcomes.

Assumptions: Control 40%, Treatment 50% (Δ = +10%); two-sided *α* = 0.05; power 80%; and dropout 10%.

Unpooled normal approximation: n_per_arm = ((1.96 + 0.84)^2^ × [0.45 × 0.55 + 0.55 × 0.45])/(0.10)^2^ ≈ 388 per arm predropout. Inflated by 10% → 427; plan 430 per arm (860 total) for conservative rounding and operational ease. Consider +3%–5% if center heterogeneity or attrition >10%.

#### Operational considerations

Randomization will be stratified by APHRODITE group to improve treatment balance and precision of subgroup estimates. Baseline characteristics will be presented by the treatment arm within each APHRODITE group, and subgroup-specific effect estimates will be included in the main results table.

### Efficacy

#### Primary efficacy analysis

The primary efficacy endpoint of the trial is the proportion of participants who achieve sperm availability suitable for ICSI at any time from randomization through Week 16. Success is defined as the presence of viable sperm obtained either through ejaculated semen or through micro-TESE. All sperm-availability determinations will be centrally reviewed and adjudicated by laboratory personnel who remain blinded to treatment allocation. The endpoint is evaluated under a treatment-policy framework, meaning all participants are analyzed according to their randomized group regardless of treatment adherence, procedures performed, or timing variations. This approach provides an unbiased estimate of the overall effectiveness of hormonal therapy compared with standard-of-care management.

#### Secondary efficacy endpoints

1. Micro-TESE sperm retrieval success rate (SSR) among those undergoing surgery.
2. Need for surgical sperm retrieval (any micro-TESE performed by Week 16).
3. Time to first sperm availability (ejaculation or micro-TESE), from randomization.
4. Embryological outcomes: ICSI fertilization rate, blastulation rate, blastocyst quality, and top-1-quality blastocyst rate (per couple cycle).
5. Reproductive outcomes: Clinical pregnancy and miscarriage rates per embryo transfer; live birth (exploratory).

#### Efficacy analysis

- **Primary analysis:** Compare proportions at Week 16 between arms using a risk difference and risk ratio with 95% CIs (Wald/Newcombe). The primary efficacy analysis will use a mixed-effects generalized linear model including a treatment arm and the APHRODITE group as fixed effects and the study site as a random intercept to account for clustering by center. A log-binomial mixed model will be preferred for estimating adjusted risk ratios. If convergence issues occur, a modified Poisson mixed-effects model with robust variance will be used as a prespecified alternative. The modified Poisson approach is specified solely as a fallback method in the event of log-binomial model non-convergence and does not represent a separate primary analytical method.
- **Sensitivity analyses:**
  • **Time**-**to**-**event** (Cox model; log-rank) for time to first sperm availability.
  • **Per**-**protocol** analysis excluding major deviations.
  • **Tipping**-**point** analyses for missing outcomes.
- **Subgroups (prespecified):** APHRODITE Group 3 vs. 4, site, baseline TT strata (<250 vs. 250–349 ng/dL), age (<35 vs. ≥35), varicocele grade (<3 vs. 3). Interaction terms to assess heterogeneity.
- **Multiplicity:** Secondary endpoints reported with 95% CIs; no formal alpha allocation unless specified in a hierarchical plan.
- **Handling intercurrent events:** Treatment policy (analyze as randomized). For couples without ART attempts by Week 16, embryologic/reproductive endpoints treated as not evaluable; no imputation for primary endpoint beyond the landmark rule.

#### Randomization and masking

Randomization: Central web platform; 1:1 allocation; stratified by site and APHRODITE group (3 and 4); variable block sizes; concealed via pharmacy/Clinical Research coordinator (CRC)-controlled access.

Masking: Surgeons/participants unblinded; laboratory personnel and central adjudicators blinded. Embryologists adjudicate outcomes using deidentified labels.

## Discussion

### Generalizability

The results of this trial can be applied to a wide range of men with idiopathic non-obstructive azoospermia who also have low testosterone and elevated FSH, as these features are commonly seen in daily infertility practice. By including participants from multiple centers and using criteria that reflect real-world presentations, this study captures a representative group of patients. The standardized protocol for hormonal treatment, semen testing, and surgical procedures further strengthens the ability to apply the findings to similar clinical settings where male infertility is managed.

The difference in procedural timing between study arms reflects the pragmatic nature of the trial design. The control arm follows standard clinical practice without delaying indicated surgery, whereas the intervention arm incorporates a predefined hormonal optimization period intended to encompass approximately one spermatogenic cycle before surgical sperm retrieval is considered.

The composite primary endpoint was intentionally designed to reflect a pragmatic and patient-centered treatment goal: achieving sperm availability for ICSI irrespective of whether sperm is obtained from ejaculation or surgery. Although these represent distinct clinical pathways, both outcomes are biologically linked to spermatogenic recovery and are clinically meaningful to couples pursuing assisted reproduction. Importantly, the protocol prospectively separates micro-TESE avoidance and surgical sperm retrieval success as secondary endpoints to allow clinically interpretable assessment of these distinct components.

### Expected impact of the proposed trial

This study is expected to provide important evidence on whether hormonal therapy before micro-TESE can improve sperm availability in men with idiopathic NOA. If the intervention proves beneficial, it may decrease the need for surgery, increase sperm retrieval success, and support better reproductive outcomes for patients and their partners. Even if no added benefit is found, the trial will offer high-quality data that can help clinicians avoid ineffective or unnecessary treatments. Overall, this study has the potential to influence future clinical practice, improve counseling, and guide more personalized care for men with NOA.

### Dissemination plan

The findings of the trial will be shared through publication in peer-reviewed scientific journals and presentations at national and international meetings in reproductive medicine and urology. Summaries will also be provided to participating centers and made available to enrolled participants in clear, understandable language. All results will be reported honestly and transparently, without revealing individual participant identities. The study team may also use the findings to contribute to educational materials or future guideline updates.

### Limitations

Several limitations of this study should be recognized. Participants and surgeons cannot be blinded to the treatment they receive, which may influence decisions around the timing of micro-TESE. Differences in surgical experience across centers may introduce variability, although a centralized review of outcomes helps reduce this effect. The 16-week treatment period may not capture longer-term responses to hormonal therapy. In addition, the study excludes men with genetic causes of NOA and those with normal testosterone levels, and therefore, the findings may not apply to these groups.

The difference in the timing of micro-TESE between study arms may be perceived as a limitation, but it actually reflects the pragmatic nature of the protocol rather than differential access to treatment. The control arm follows routine clinical scheduling, whereas the intervention arm incorporates a biologically motivated hormonal optimization period approximating one spermatogenic cycle and allowing endocrine titration before surgery. Multiple sensitivity analyses and treatment-policy estimand methodology were prespecified to minimize potential timing-related bias.

Idiopathic NOA is biologically heterogeneous, and the present protocol intentionally focuses on a selected subgroup characterized by biochemical hypogonadism and elevated FSH levels consistent with APHRODITE Groups 3–4. Consequently, the findings may not be generalizable to men with normogonadal NOA, defined genetic etiologies, prior gonadotoxic exposure, or other clinical phenotypes commonly encountered in practice. These restrictions were intentionally implemented to enrich the study population for individuals in whom hormonal modulation would be physiologically justified.

## Estimands (ICH E9[R1])

Primary estimand (treatment-policy):

- Treatment conditions: Arm A = hCG + FSH regimen with monthly hormone-guided titration (FSH fixed 150 IU 2×/week; increase to 150 IU 3×/week if “FSH reset” < 1.5 IU/L); Arm B = standard care (no gonadotropins).
- Population: All randomized idiopathic NOA participants meeting eligibility (TT < 350 ng/dL on two fasting mornings; FSH ≥ 7.6 IU/L; APHRODITE Groups 3 or 4).
- Variable (endpoint): Sperm available for ICSI by 16 weeks—success if sperm is present in ejaculate at Week 12 or 16, or (if ejaculate is negative) sperm retrieved at micro-TESE at Week 16.
- Intercurrent events strategy: Treatment-policy—outcomes counted regardless of adherence or pathway (e.g., early stop, skipping micro-TESE after ejaculate success, elective early micro-TESE at 12 weeks). Missing primary outcomes are treated as failure in the primary analysis, with supportive hypothetical analyses.
- Summary measure: Proportion with success by 16 weeks; between-arm comparison via risk ratio and risk difference with 95% CIs using a mixed-effects model (fixed: arm, APHRODITE group; random: site).

Supportive estimands:

- Hypothetical estimand: “What would the outcomes be if all participants completed week-16 assessments?”—analyzed using multiple imputation under MAR with delta-based MNAR sensitivity.
- Per-protocol estimand: Effect among participants without major protocol deviations (e.g., prohibited concomitants, major non-adherence, early elective micro-TESE without week-16 assessment).
