# Supplementary material for: Gonadotropin therapy in idiopathic hypogonadal non-obstructive azoospermia (APHRODITE Groups 3–4): a multicenter randomized controlled trial
Source: Front Reprod Health. 2026 Jul 9;8:1867412. doi: 10.3389/frph.2026.1867412 (PMC13391572; doi:10.3389/frph.2026.1867412)
Supplement: Supplementary file 1 [file Table1.docx]

**Dummy Tables — Intention-to-Treat (ITT)**

Table I. Baseline characteristics (by randomized arm)

| Characteristic | Arm A (hCG + FSH) | Arm B (Control) | Total |
| --- | --- | --- | --- |
| N | … | … | … |
| Age (years) | … | … | … |
| BMI (kg/m²) | … | … | … |
| Duration of infertility (years) | … | … | … |
| Etiology: idiopathic (n, %) | … | … | … |
| FSH (IU/L) | … | … | … |
| LH (IU/L) | … | … | … |
| Estradiol (pmol/L) | … | … | … |
| Total testosterone (ng/dL) | … | … | … |
| Testicular volume (mL) | … | … | … |
| Other (specify) | … | … | … |

Table II. Sperm available for ICSI by week 16 (ITT) — overall cohort

|  | Arm A | Arm B | Risk Ratio (95% CI) | Adjusted Risk Ratio (95% CI)* |
| --- | --- | --- | --- | --- |
| N randomized | … | … | … | … |
| Events, n (%) | … | … | … | … |

*Adjusted for site (random) and APHRODITE (fixed)

Table III. Sperm available for ICSI — APHRODITE Group 3 (ITT)

|  | Arm A | Arm B | Risk Ratio (95% CI) | Adjusted Risk Ratio (95% CI)* |
| --- | --- | --- | --- | --- |
| N randomized | … | … | … | … |
| Events, n (%) | … | … | … | … |

Table IV. Sperm available for ICSI — APHRODITE Group 4 (ITT)

|  | Arm A | Arm B | Risk Ratio (95% CI) | Adjusted Risk Ratio (95% CI)* |
| --- | --- | --- | --- | --- |
| N randomized | … | … | … | … |
| Events, n (%) | … | … | … | … |

Table V. Adverse events (Safety population)

| Adverse Event | Arm A (n, %) | Arm B (n, %) | Grade / Notes |
| --- | --- | --- | --- |
| Any AE | … | … | … |
| Serious AE | … | … | … |
| Discontinuation due to AE | … | … | … |
| Gynecomastia | … | … | … |
| Injection-site reaction | … | … | … |
| Mood/energy change | … | … | … |
| Lab abnormalities (E2, Hct) | … | … | … |

**Per-Protocol (PP) — Dummy Tables**

Table II-PP. Sperm available for ICSI by week 16 (PP) — overall cohort

|  | Arm A (PP) | Arm B (PP) | Risk Ratio (95% CI) | Adjusted Risk Ratio (95% CI)* |
| --- | --- | --- | --- | --- |
| N (PP set) | … | … | … | … |
| Events, n (%) | … | … | … | … |

*Adjusted for site (random) and APHRODITE (fixed)

Table III-PP. Sperm available for ICSI — APHRODITE Group 3 (PP)

|  | Arm A (PP) | Arm B (PP) | Risk Ratio (95% CI) | Adjusted Risk Ratio (95% CI)* |
| --- | --- | --- | --- | --- |
| N (PP set) | … | … | … | … |
| Events, n (%) | … | … | … | … |

Table IV-PP. Sperm available for ICSI — APHRODITE Group 4 (PP)

|  | Arm A (PP) | Arm B (PP) | Risk Ratio (95% CI) | Adjusted Risk Ratio (95% CI)* |
| --- | --- | --- | --- | --- |
| N (PP set) | … | … | … | … |
| Events, n (%) | … | … | … | … |

**Figure 1. CONSORT Flow Diagram (editable table template)**

| Assessed for eligibility (n = …) | |
| --- | --- |
| Randomized (n = …) | |
| Allocated to Arm A: hCG + FSH (n = …)  • Received allocated intervention (n = …)  • Did not receive (reasons) (n = …) | Allocated to Arm B: Control (n = …)  • Received allocated intervention (n = …)  • Did not receive (reasons) (n = …) |
| Follow-up Arm A  • Lost to follow-up (reasons) (n = …)  • Discontinued intervention (reasons) (n = …) | Follow-up Arm B  • Lost to follow-up (reasons) (n = …)  • Discontinued (reasons) (n = …) |
| Assessment Arm A  • Week 12 semen  • Week 16 semen  • Micro-TESE if needed | Assessment Arm B  • Day-of-surgery semen  • Micro-TESE if needed |
| Analyzed (ITT) Arm A (n = …)  • Excluded (reasons) (n = …) | Analyzed (ITT) Arm B (n = …)  • Excluded (reasons) (n = …) |
| Primary outcome: Sperm available for ICSI by week 16 (composite) | |
| Secondary outcomes and analyses (see sections) | |

**Appendices: DSMB, Randomization & Masking SOPs, and Analysis Sets**

**I. Appendix A. Randomization & Masking — Practical SOPs**

Design

- Allocation: 1:1.
- Strata: site and APHRODITE group (3 vs 4).
- Blocks: variable sizes (e.g., 4 and 6), randomly mixed.

Sequence generation & concealment

- Independent statisticians generate the stratified, variable-block sequence.
- Load into a centralized system (e.g., REDCap Randomization Module, OpenClinica Randomize, or Sealed Envelope/IWRS).
- Sites randomize after eligibility confirmation; allocation concealed until assignment.

Roles

- Independent statistician: sequence generation; system setup/admin.
- Unblinded pharmacy: views assignment; prepares/dispenses hCG + FSH per protocol.
- Investigators/Surgeons: enroll, treat, schedule procedures (unblinded).
- Lab/Embryology/Central adjudicators: blinded; receive de-identified samples labeled with StudyID only.

Workflow (site perspective)

- Screen and confirm APHRODITE group (3: FSH 7.6–12.0 IU/L; 4: >12.0 IU/L) and hypogonadism (TT <350 ng/dL on two fasting mornings).
- Enroll in eCRF → generate StudyID.
- Randomize via IWRS/REDCap with strata = site and group.
- Pharmacy notified (system) → dispenses per arm (Arm A: hCG + FSH).
- Monthly titration labs (TT/E2/FSH/LH) restricted to the clinical team; embryology/adjudication teams remain blinded and receive only sperm presence/absence outputs.
- Unblinding pathway (if necessary for safety): documented, sponsor-controlled, and logged.

Fallback (if system unavailable)

- SNOSE (Sequentially Numbered, Opaque, Sealed Envelopes) prepared by the independent statistician; tamper-evident; stored and issued by pharmacy with sign-out log.

Blinding safeguards

- Separate data streams (titration labs vs outcome adjudication).
- Lab requisitions: StudyID only; no arm or treatment hints.
- eCRF permissions: outcome adjudicators have read-only access to outcome pages; no access to treatment pages.
- Monitoring: periodic checks that blinding is intact; staff retraining as needed.

**Appendix B. Analysis Sets and Per-Protocol Criteria**

Intention-to-Treat (ITT)

- All randomized participants analyzed as assigned.
- Primary estimand = treatment-policy up to week 16 (success if sperm in ejaculate at week 12/16 or, if ejaculate negative, at week-16 micro-TESE).
- Missing primary outcomes imputed as failure (conservative).

Per-Protocol (PP) — supportive only

- Exclude major deviations:
- Elective early micro-TESE at 12 weeks without a week-16 assessment.
- Prohibited concomitant medications or off-protocol hormonal therapy.
- Critical non-adherence to dosing/titration schedule (e.g., persistent failure to attend monthly hormone checks or to take assigned hCG + FSH).
- Other pre-specified violations materially affecting primary outcome ascertainment.

Notes

- PP results do not replace ITT; they provide robustness checks.
- Subgroup PP analyses for APHRODITE Group 3 and Group 4 mirror ITT subgroup tables.

**Appendix C. Data and Safety Monitoring Board (DSMB)**

Definition & independence

- An independent committee that monitors participant safety, data quality, protocol adherence, and overall trial conduct.
- The DSMB may recommend continuation, modification, pause, or termination.

Composition (3–5 members)

- Urology/andrology clinician
- Reproductive endocrinology/IVF clinician
- Independent statistician (chair or co-chair)
- Bioethicist and/or patient advocate

Cadence

- Safety-only review at ~50% randomized with ≥12-week follow-up available.
- Ad-hoc meetings if safety triggers are met.

Charter contents

- Scope and decision authority; membership and conflicts of interest.
- Meeting timelines and quorum.
- Safety signal thresholds (e.g., unexpected SAEs; clinically meaningful lab abnormalities such as persistent hematocrit elevation).
- Data package specification (see below).
- Communication timelines to sponsor/PI and IRB/EC.
- Decision framework (continue / modify / pause / stop).

Interim data package (minimum)

- Enrollment and retention by site and APHRODITE group (3 vs 4).
- Protocol deviations (e.g., early elective micro-TESE at 12 weeks).
- Safety: AEs/SAEs by arm; laboratory trends (E2, hematocrit, etc.).
- Blinded summary of primary endpoint status to date (no arm-level unblinding).

Communication & actions

- DSMB recommendation letter (continue/modify/pause/stop) to sponsor/PI.
- Sponsor/PI documents action taken; IRB/EC notified per local policy.
- Blinding preserved for outcomes team unless unblinding is essential for safety.

**Appendix D.** **Estimands (ICH E9[R1])**

Primary estimand (treatment-policy):

- Treatment conditions: Arm A = hCG + FSH regimen with monthly hormone-guided titration (FSH fixed 150 IU 2×/week; increase to 150 IU 3×/week if 'FSH reset' <1.5 IU/L); Arm B = standard care (no gonadotropins).
- Population: All randomized idiopathic NOA participants meeting eligibility (TT <350 ng/dL on two fasting mornings; FSH ≥7.6 IU/L; APHRODITE Groups 3 or 4).
- Variable (endpoint): Sperm available for ICSI by 16 weeks — success if sperm is present in ejaculate at week 12 or 16, or (if ejaculate is negative) sperm retrieved at micro-TESE at week 16.
- Intercurrent events strategy: Treatment-policy — outcomes counted regardless of adherence or pathway (e.g., early stop, skipping micro-TESE after ejaculate success, elective early micro-TESE at 12 weeks). Missing primary outcomes are treated as failure in the primary analysis, with supportive hypothetical analyses.
- Summary measure: Proportion with success by 16 weeks; between-arm comparison via risk ratio and risk difference with 95% CIs using a mixed-effects model (fixed: arm, APHRODITE group; random: site).

Supportive estimands:

- Hypothetical estimand: ‘What would the outcomes be if all participants completed week-16 assessments?’ — analyzed using multiple imputation under MAR with delta-based MNAR sensitivity.
- Per-protocol estimand: Effect among participants without major protocol deviations (e.g., prohibited concomitants, major non-adherence, early elective micro-TESE without week-16 assessment).

**CHECK-LIST**

**Randomization & Concealment SOP**

- Independent statistician generates stratified (site, APHRODITE 3/4), variable-block list; uploads to IWRS/REDCap.
- User roles set: PI (randomize), pharmacy (view assignment), coordinators (screen/enroll), lab/adjudicators (no arm access).
- Test run + audit trail verification before first patient.
- SNOSE envelopes prepared as emergency backup; stored in pharmacy; sign-out log maintained.

**Masking SOP**

- Embryology and adjudication teams trained as blinded.
- Samples labeled StudyID only; requisitions scrubbed of treatment info.
- eCRF permissions restricted; outcome pages segregated.
- Unblinding pathway documented (should be unnecessary).

**DSMB Charter (key bullets)**

- Membership, independence, conflicts.
- Meeting timing (50% information).
- Safety triggers (e.g., SAE patterns; hematocrit thresholds).
- Decision framework and communication timelines.
